# 2-Amino-4-(4-meth­oxy­phen­yl)-5-oxo-4*H*,5*H*-pyrano[3,2-*c*]chromene-3-carbo­nitrile acetic acid monosolvate

**DOI:** 10.1107/S2414314623005588

**Published:** 2023-06-30

**Authors:** Bimal Bhushan Chakraborty, Saurav Paul, Siddique Anwar, Sudip Choudhury

**Affiliations:** a Department of Chemistry, Assam University, Silchar 788011, India; b Centre for Soft Matter, Department of Chemistry, Assam University, Silchar 788011, India; University of Aberdeen, United Kingdom

**Keywords:** crystal structure, co-crystal, acetic acid dimer, chromene, carbonitrile

## Abstract

In the title co-crystal, C_20_H_14_N_2_O_4_·C_2_H_4_O_2_, the expected proton transfer from acetic acid to amine has not occurred. In the crystal, the chromene mol­ecules are linked by N—H⋯O and N—H⋯N hydrogen bonds to generate [100] columns. The acetic acid mol­ecules form inversion dimers linked by pairwise O—H⋯O hydrogen bonds and occupy voids between the columns.

## Abstract 2

The crystal structure of the title compound contains the host mol­ecule and acetic acid in 1:1 ratio without undergoing salt formation.

## Graphical abstract

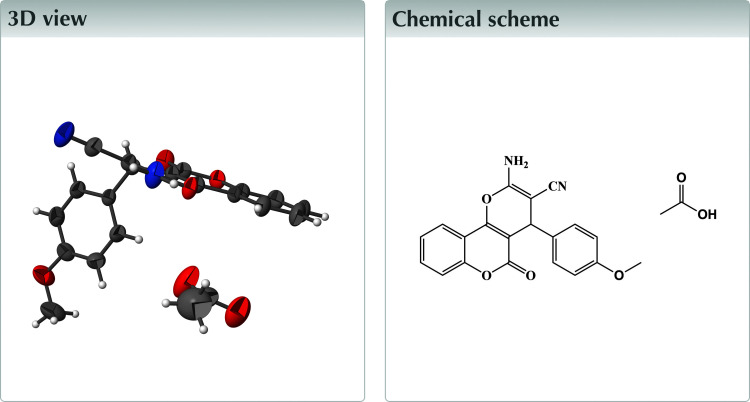

## Structure description

Pyrano[3,2-*c*]chromene derivatives enjoy attention from researchers due to their pharmacological activity (Siziani *et al.*, 2022 ▸; Tashrifi *et al.*, 2020 ▸), heavy metal chemisensing (Mohajer *et al.*, 2022 ▸), semiconductivity (Mal *et al.*, 2022 ▸), *etc*. As part of our studies in this area, the crystal structure of the 1:1 co-crystal of 2-amino-4-(4-meth­oxy­phen­yl)-5-oxo-4*H*,5*H*-pyrano[3,2-*c*]chromene-3-carbo­nitrile and acetic acid is now reported. The compound was crystallized from acetic acid, but the expected proton transfer from the carb­oxy­lic acid to the amine group did not occur.

**Figure 1:**
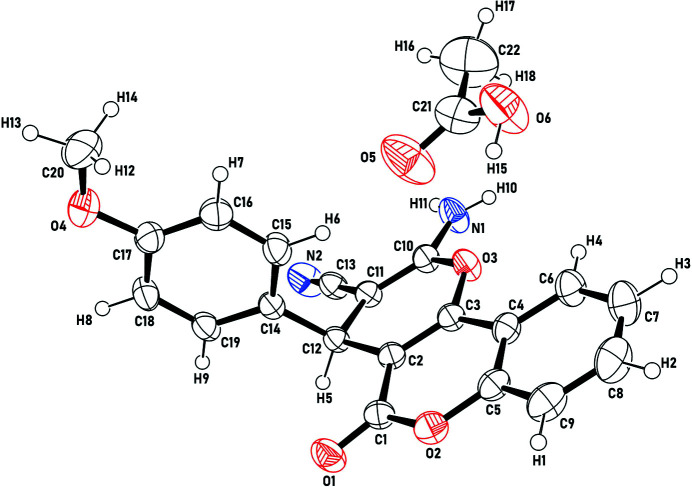
Molecular structure of the title compound with displacement ellipsoids drawn at the 50% probability level.

The title compound crystallizes in the triclinic space group *P* 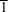 with one pyrano[3,2-*c*]chromene mol­ecule and one acetic acid mol­ecule in the asymmetric unit (Fig. 1 ▸). Unexpectedly, although crystallized from a solvent of glacial acetic acid, the –NH_2_ group present in the pyran­ochromene framework was not protonated. The dihedral angle between the planes of the C1–C12/O2/O3 fused ring (r.m.s. deviation = 0.079 Å) and the pendant C14–C19 ring is 89.00 (6)°, and the C atom of the meth­oxy substituent deviates by 0.132 (2) Å from its attached ring.

**Figure 2:**
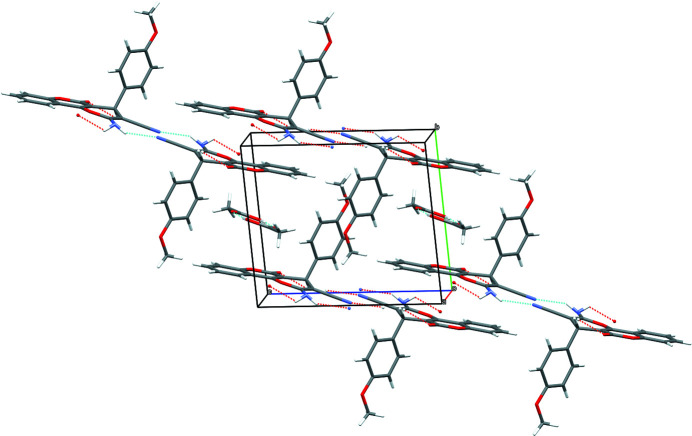
Packing arrangement of the title compound. Hydrogen bonds are shown as dotted lines.

In the crystal, the pyrano[3,2-*c*]chromene mol­ecules are linked by N1—H11⋯N2^i^ hydrogen bonds (Table 1 ▸) to generate centrosymmetric 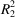 (12) loops and the dimers are linked into [100] chains by N1—H10⋯O1^ii^ links to generate [100] columns. The acetic acid mol­ecules maintain their hydrogen-bonded dimeric form (*via* pairwise O6—H15⋯O5^iii^ links) without any directional inter­actions with the pyrano[3,2-*c*]chromene columns (Fig. 2 ▸). The acetic acid dimers occupy the space between pyran­ochromene columns (about 7.4 Å) and are positioned approximately parallel to the pyran­ochromene plane of the host mol­ecule; a weak C15—H6⋯O5 hydrogen bond occurs between host and guest. The significant difference between the lengths of the C21—O5 [1.197 (3) Å] and C21—O6 [1.284 (3) Å] bonds infers that the acetic acid mol­ecule remains in its protonated state.

## Synthesis and crystallization

4-Hy­droxy­coumarin or 4-hy­droxy-2*H*-benzo[*h*]chromen-2-one (1.00 mmol), 4-meth­oxy­benzaldehyde (1.00 mmol), malono­nitrile (1.00 mmol) and catalyst DABCO (10 mol%) were ground with a mortar and pestle for about 10 min. Upon completion of the reaction, the product was washed several times with ethanol to get the pure product, a white solid. The purity of the compound was confirmed by fluorescent HPTLC (Merck) and melting point (observed 238°C, reported 237°C; Shaabani *et al.*, 2007 ▸). FT–IR (KBr, cm^−1^): 3360, 3184, 2980, 1726, 1596, 1462; ^1^H NMR (400 MHz, DMSO-*d*_6_): 3.73 (*s*, 3H), 4.40 (*s*, 1H), 6.89 (*d*, 2H, *J* = 8.8 Hz, 7.19 (*d*, 2H, *J* = 8.4 Hz), 7.35 (*s*, 2H), 7.52–7.40 (*m*, 2H), 7.73 (*dt*, 1H, *J* = 8.8, 1.6 Hz), 7.92 (*dd*, *J* = 8.0, 1.6 Hz); ^13^C NMR (100 MHz, DMSO-*d*_6_): 36.1, 55.0, 58.3, 104.3, 112.9, 113.9, 116.5, 119.2, 122.4, 124.6, 128.7, 132.8, 135.4, 152.1, 153.1, 157.9, 158.3, 159.5. Suitable crystals of the title compound were grown by dissolving the compound in glacial acetic acid. The solution was kept undisturbed for a period of two weeks in an NMR tube (OD 5 mm) and the grown crystals were carefully recovered and washed with hexane and dried.

## Refinement

Crystal data, data collection and structure refinement details are summarized in Table 2 ▸.

## Supplementary Material

Crystal structure: contains datablock(s) I. DOI: 10.1107/S2414314623005588/hb4427sup1.cif

Supporting information file. DOI: 10.1107/S2414314623005588/hb4427Isup3.mol

CCDC reference: 2271965

Additional supporting information:  crystallographic information; 3D view; checkCIF report

## Figures and Tables

**Table 1:** Hydrogen-bond geometry (Å, °)

*D*—H⋯*A*	*D*—H	H⋯*A*	*D*⋯*A*	*D*—H⋯*A*
N1—H11⋯N2^i^	0.85 (2)	2.22 (2)	3.062 (2)	172 (2)
N1—H10⋯O1^ii^	0.81 (2)	2.31 (2)	3.111 (2)	168 (2)
O6—H15⋯O5^iii^	1.00 (4)	1.67 (4)	2.664 (3)	172 (3)
C15—H6⋯O5	0.93	2.39	3.251 (2)	154

Symmetry codes: (i) 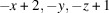 ; (ii) 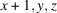 ; (iii) 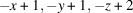 .

**Table 2:** Experimental details

Crystal data	
Chemical formula	C_20_H_14_N_2_O_4_·C_2_H_4_O_2_
*M*_r_	406.38
Crystal system, space group	Triclinic, *P* 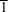
Temperature (K)	296
*a*, *b*, *c* (Å)	7.9303 (6), 11.2977 (9), 11.9988 (9)
α, β, γ (°)	82.468 (4), 77.379 (4), 73.419 (4)
*V* (Å^3^)	1002.71 (14)
*Z*	2
Radiation type	Mo *K*α
μ (mm^−1^)	0.10
Crystal size (mm)	0.36 × 0.36 × 0.30

Data collection	
Diffractometer	Bruker APEXII CCD
No. of measured, independent and observed [*I* > 2σ(*I*)] reflections	17101, 4807, 3457
*R*_int_	0.025
(sin θ/λ)_max_ (Å^−1^)	0.662

Refinement	
*R*[*F* ^2^ > 2σ(*F* ^2^)], *wR*(*F* ^2^), *S*	0.050, 0.157, 1.06
No. of reflections	4807
No. of parameters	285
H-atom treatment	H atoms treated by a mixture of independent and constrained refinement
Δρ_max_, Δρ_min_ (e Å^−3^)	0.30, −0.25

Computer programs: *APEX2* and *SAINT* (Bruker 2015 ▸), *SHELXT2018* (Sheldrick, 2015*a* ▸), *SHELXL2018* (Sheldrick, 2015*b* ▸) and *OLEX2* (Dolomanov *et al.*, 2009 ▸; Bourhis *et al.*, 2015 ▸).

## full crystallographic data

### Crystal data

**Table:**

C_20_H_14_N_2_O_4_·C_2_H_4_O_2_	*F*(000) = 424
*M**_r_* = 406.38	*D*_x_ = 1.346 Mg m^−^^3^
Triclinic, *P*1	Melting point: 511.15 K
*a* = 7.9303 (6) Å	Mo *K*α radiation, λ = 0.71073 Å
*b* = 11.2977 (9) Å	Cell parameters from 5371 reflections
*c* = 11.9988 (9) Å	θ = 2.7–27.9°
α = 82.468 (4)°	µ = 0.10 mm^−^^1^
β = 77.379 (4)°	*T* = 296 K
γ = 73.419 (4)°	Block, white
*V* = 1002.71 (14) Å^3^	0.36 × 0.36 × 0.30 mm
*Z* = 2	

### Data collection

**Table:**

Bruker APEXII CCD diffractometer	3457 reflections with *I* > 2σ(*I*)
Radiation source: sealed tube	*R*_int_ = 0.025
Graphite monochromator	θ_max_ = 28.1°, θ_min_ = 2.7°
φ and ω scans	*h* = −10→10
17101 measured reflections	*k* = −14→14
4807 independent reflections	*l* = −15→15

### Refinement

**Table:**

Refinement on *F*^2^	Hydrogen site location: mixed
Least-squares matrix: full	H atoms treated by a mixture of independent and constrained refinement
*R*[*F*^2^ > 2σ(*F*^2^)] = 0.050	*w* = 1/[σ^2^(*F*_o_^2^) + (0.0858*P*)^2^ + 0.1068*P*] where *P* = (*F*_o_^2^ + 2*F*_c_^2^)/3
*wR*(*F*^2^) = 0.157	(Δ/σ)_max_ < 0.001
*S* = 1.06	Δρ_max_ = 0.30 e Å^−^^3^
4807 reflections	Δρ_min_ = −0.24 e Å^−^^3^
285 parameters	Extinction correction: SHELXL2018 (Sheldrick, 2015*b*), Fc^*^=kFc[1+0.001xFc^2^λ^3^/sin(2θ)]^-1/4^
0 restraints	Extinction coefficient: 0.037 (6)
Primary atom site location: iterative	

### Special details

**Table:**

Geometry. All esds (except the esd in the dihedral angle between two l.s. planes) are estimated using the full covariance matrix. The cell esds are taken into account individually in the estimation of esds in distances, angles and torsion angles; correlations between esds in cell parameters are only used when they are defined by crystal symmetry. An approximate (isotropic) treatment of cell esds is used for estimating esds involving l.s. planes.

### Fractional atomic coordinates and isotropic or equivalent isotropic displacement parameters (Å^2^)

**Table:**

	*x*	*y*	*z*	*U*_iso_*/*U*_eq_	
C1	0.28503 (19)	0.15224 (13)	0.94770 (12)	0.0398 (3)	
C2	0.46268 (18)	0.13992 (12)	0.87929 (11)	0.0343 (3)	
C3	0.59600 (18)	0.14624 (12)	0.92882 (11)	0.0351 (3)	
C4	0.5684 (2)	0.16975 (12)	1.04776 (11)	0.0388 (3)	
C5	0.3950 (2)	0.18548 (13)	1.10966 (11)	0.0416 (3)	
C6	0.7039 (2)	0.17741 (15)	1.10147 (13)	0.0506 (4)	
H4	0.820649	0.167223	1.060769	0.061*	
C7	0.6624 (3)	0.20027 (18)	1.21552 (15)	0.0649 (5)	
H3	0.752115	0.204468	1.252281	0.078*	
C8	0.4892 (3)	0.21697 (18)	1.27573 (14)	0.0662 (5)	
H2	0.463455	0.233314	1.352487	0.079*	
C9	0.3539 (3)	0.20992 (16)	1.22439 (13)	0.0566 (4)	
H1	0.237323	0.221273	1.265566	0.068*	
C10	0.81505 (19)	0.09187 (13)	0.76421 (11)	0.0372 (3)	
C11	0.69033 (18)	0.08117 (12)	0.70792 (11)	0.0350 (3)	
C12	0.49036 (17)	0.12492 (12)	0.75303 (10)	0.0339 (3)	
H5	0.434598	0.060982	0.742969	0.041*	
C13	0.74784 (19)	0.03627 (13)	0.59735 (12)	0.0414 (3)	
C14	0.40507 (17)	0.24512 (12)	0.68869 (10)	0.0345 (3)	
C15	0.4317 (2)	0.35587 (13)	0.70738 (13)	0.0471 (4)	
H6	0.499634	0.356797	0.761194	0.056*	
C16	0.3594 (2)	0.46567 (14)	0.64780 (14)	0.0506 (4)	
H7	0.378473	0.539410	0.661815	0.061*	
C17	0.2593 (2)	0.46490 (14)	0.56784 (13)	0.0478 (4)	
C18	0.2344 (2)	0.35441 (16)	0.54666 (14)	0.0506 (4)	
H8	0.168538	0.353381	0.491619	0.061*	
C19	0.3064 (2)	0.24558 (14)	0.60666 (12)	0.0423 (3)	
H9	0.288535	0.171768	0.591792	0.051*	
C20	0.1897 (3)	0.68441 (18)	0.5279 (2)	0.0792 (6)	
H12	0.134892	0.699323	0.606260	0.119*	
H13	0.127714	0.747593	0.478338	0.119*	
H14	0.312930	0.685832	0.515177	0.119*	
N1	0.99246 (18)	0.06727 (16)	0.73010 (13)	0.0548 (4)	
H11	1.045 (3)	0.044 (2)	0.664 (2)	0.082*	
H10	1.050 (3)	0.0795 (18)	0.7738 (17)	0.063 (6)*	
N2	0.7914 (2)	0.00034 (16)	0.50810 (12)	0.0636 (4)	
O1	0.15733 (14)	0.14244 (11)	0.91391 (10)	0.0554 (3)	
O2	0.25831 (14)	0.17565 (10)	1.06096 (8)	0.0469 (3)	
O3	0.76834 (13)	0.13240 (10)	0.87243 (8)	0.0438 (3)	
O4	0.18023 (18)	0.56733 (12)	0.50445 (12)	0.0718 (4)	
C21	0.7402 (3)	0.47407 (18)	0.9117 (2)	0.0699 (5)	
C22	0.9237 (4)	0.4564 (3)	0.8413 (3)	0.1178 (10)	
H16	0.917608	0.462359	0.761649	0.177*	
H17	0.974923	0.519298	0.855137	0.177*	
H18	0.997116	0.376303	0.861655	0.177*	
O5	0.6192 (2)	0.4549 (2)	0.87859 (14)	0.1048 (6)	
O6	0.7183 (3)	0.51493 (19)	1.01024 (16)	0.0988 (6)	
H15	0.594 (5)	0.518 (3)	1.052 (3)	0.139 (11)*	

### Atomic displacement parameters (Å^2^)

**Table:**

	*U*^11^	*U*^22^	*U*^33^	*U*^12^	*U*^13^	*U*^23^
C1	0.0345 (7)	0.0454 (7)	0.0371 (7)	−0.0108 (6)	−0.0012 (6)	−0.0037 (5)
C2	0.0324 (7)	0.0380 (6)	0.0312 (6)	−0.0091 (5)	−0.0030 (5)	−0.0035 (5)
C3	0.0335 (7)	0.0403 (7)	0.0302 (6)	−0.0096 (6)	−0.0023 (5)	−0.0047 (5)
C4	0.0451 (8)	0.0401 (7)	0.0304 (6)	−0.0095 (6)	−0.0066 (6)	−0.0051 (5)
C5	0.0478 (9)	0.0398 (7)	0.0337 (7)	−0.0098 (6)	−0.0024 (6)	−0.0032 (5)
C6	0.0531 (10)	0.0607 (9)	0.0406 (8)	−0.0132 (8)	−0.0120 (7)	−0.0119 (7)
C7	0.0770 (13)	0.0790 (12)	0.0458 (9)	−0.0182 (10)	−0.0217 (9)	−0.0178 (8)
C8	0.0889 (15)	0.0741 (11)	0.0341 (8)	−0.0165 (11)	−0.0079 (9)	−0.0174 (8)
C9	0.0674 (11)	0.0580 (9)	0.0369 (7)	−0.0129 (8)	0.0051 (7)	−0.0104 (7)
C10	0.0343 (7)	0.0459 (7)	0.0304 (6)	−0.0098 (6)	−0.0025 (5)	−0.0073 (5)
C11	0.0342 (7)	0.0404 (7)	0.0303 (6)	−0.0107 (6)	−0.0018 (5)	−0.0072 (5)
C12	0.0321 (7)	0.0398 (6)	0.0321 (6)	−0.0123 (6)	−0.0054 (5)	−0.0060 (5)
C13	0.0351 (8)	0.0517 (8)	0.0395 (7)	−0.0145 (6)	−0.0022 (6)	−0.0121 (6)
C14	0.0307 (7)	0.0434 (7)	0.0301 (6)	−0.0105 (6)	−0.0044 (5)	−0.0058 (5)
C15	0.0541 (9)	0.0471 (8)	0.0470 (8)	−0.0148 (7)	−0.0214 (7)	−0.0051 (6)
C16	0.0561 (10)	0.0423 (7)	0.0578 (9)	−0.0149 (7)	−0.0172 (8)	−0.0039 (6)
C17	0.0379 (8)	0.0505 (8)	0.0515 (8)	−0.0089 (7)	−0.0104 (7)	0.0051 (7)
C18	0.0455 (9)	0.0646 (10)	0.0477 (8)	−0.0164 (8)	−0.0222 (7)	0.0009 (7)
C19	0.0402 (8)	0.0517 (8)	0.0412 (7)	−0.0179 (7)	−0.0113 (6)	−0.0064 (6)
C20	0.0751 (14)	0.0545 (10)	0.1034 (16)	−0.0150 (10)	−0.0250 (12)	0.0192 (10)
N1	0.0308 (7)	0.0912 (11)	0.0429 (7)	−0.0141 (7)	−0.0023 (6)	−0.0193 (7)
N2	0.0572 (9)	0.0925 (11)	0.0472 (8)	−0.0284 (8)	0.0044 (7)	−0.0316 (7)
O1	0.0353 (6)	0.0801 (8)	0.0527 (6)	−0.0193 (6)	−0.0039 (5)	−0.0101 (6)
O2	0.0403 (6)	0.0604 (6)	0.0357 (5)	−0.0130 (5)	0.0036 (4)	−0.0076 (4)
O3	0.0319 (5)	0.0684 (7)	0.0334 (5)	−0.0147 (5)	−0.0026 (4)	−0.0144 (4)
O4	0.0689 (9)	0.0603 (7)	0.0882 (9)	−0.0144 (6)	−0.0377 (7)	0.0201 (6)
C21	0.0617 (13)	0.0648 (11)	0.0844 (14)	−0.0198 (10)	−0.0065 (11)	−0.0153 (10)
C22	0.0723 (17)	0.126 (2)	0.137 (3)	−0.0156 (16)	0.0149 (16)	−0.0281 (19)
O5	0.0846 (12)	0.1625 (17)	0.0870 (11)	−0.0550 (12)	0.0017 (9)	−0.0599 (11)
O6	0.0784 (12)	0.1447 (16)	0.0913 (12)	−0.0462 (11)	−0.0156 (10)	−0.0361 (11)

### Geometric parameters (Å, º)

**Table:**

C2—C1	1.4459 (19)	C18—H8	0.9300
C2—C3	1.3435 (19)	C18—C19	1.380 (2)
C3—C4	1.4439 (18)	C19—C14	1.3834 (19)
C4—C6	1.396 (2)	C19—H9	0.9300
C5—C4	1.387 (2)	C20—H12	0.9600
C5—C9	1.389 (2)	C20—H13	0.9600
C6—H4	0.9300	C20—H14	0.9600
C7—C6	1.377 (2)	N1—C10	1.3339 (19)
C7—H3	0.9300	N1—H11	0.85 (2)
C8—C7	1.378 (3)	N1—H10	0.81 (2)
C8—H2	0.9300	N2—C13	1.1416 (18)
C9—C8	1.374 (3)	O1—C1	1.2072 (17)
C9—H1	0.9300	O2—C1	1.3776 (17)
C11—C10	1.3529 (19)	O2—C5	1.3753 (18)
C11—C13	1.4161 (18)	O3—C3	1.3608 (16)
C12—C2	1.5075 (17)	O3—C10	1.3718 (15)
C12—C11	1.5165 (18)	O4—C17	1.3691 (18)
C12—H5	0.9800	O4—C20	1.414 (2)
C14—C12	1.5253 (18)	C22—C21	1.488 (3)
C14—C15	1.380 (2)	C22—H16	0.9600
C15—H6	0.9300	C22—H17	0.9600
C15—C16	1.385 (2)	C22—H18	0.9600
C16—H7	0.9300	O5—C21	1.197 (3)
C17—C16	1.375 (2)	O6—C21	1.284 (3)
C17—C18	1.381 (2)	O6—H15	1.00 (4)
			
O1—C1—C2	125.22 (13)	C15—C14—C12	120.49 (12)
O1—C1—O2	116.85 (13)	C15—C14—C19	118.23 (13)
O2—C1—C2	117.93 (12)	C19—C14—C12	121.21 (12)
C1—C2—C12	118.49 (12)	C14—C15—H6	119.2
C3—C2—C1	119.38 (12)	C14—C15—C16	121.51 (13)
C3—C2—C12	122.09 (12)	C16—C15—H6	119.2
C2—C3—C4	122.65 (13)	C15—C16—H7	120.2
C2—C3—O3	123.72 (11)	C17—C16—C15	119.55 (14)
O3—C3—C4	113.62 (12)	C17—C16—H7	120.2
C5—C4—C3	116.32 (13)	C16—C17—C18	119.60 (14)
C5—C4—C6	119.71 (13)	O4—C17—C16	124.86 (15)
C6—C4—C3	123.98 (14)	O4—C17—C18	115.54 (14)
C4—C5—C9	120.82 (15)	C17—C18—H8	119.8
O2—C5—C4	121.65 (12)	C19—C18—C17	120.42 (13)
O2—C5—C9	117.52 (14)	C19—C18—H8	119.8
C4—C6—H4	120.5	C14—C19—H9	119.7
C7—C6—C4	119.07 (16)	C18—C19—C14	120.67 (13)
C7—C6—H4	120.5	C18—C19—H9	119.7
C6—C7—H3	119.7	H12—C20—H13	109.5
C6—C7—C8	120.64 (17)	H12—C20—H14	109.5
C8—C7—H3	119.7	H13—C20—H14	109.5
C7—C8—H2	119.4	O4—C20—H12	109.5
C9—C8—C7	121.17 (15)	O4—C20—H13	109.5
C9—C8—H2	119.4	O4—C20—H14	109.5
C5—C9—H1	120.7	C10—N1—H11	122.0 (16)
C8—C9—C5	118.59 (17)	C10—N1—H10	117.6 (14)
C8—C9—H1	120.7	H11—N1—H10	120 (2)
C11—C10—O3	121.49 (12)	C5—O2—C1	121.99 (11)
N1—C10—C11	129.17 (13)	C3—O3—C10	118.34 (11)
N1—C10—O3	109.34 (12)	C17—O4—C20	118.28 (15)
C10—C11—C12	123.19 (11)	O5—C21—C22	123.0 (2)
C10—C11—C13	118.66 (12)	O5—C21—O6	121.7 (2)
C13—C11—C12	117.94 (12)	O6—C21—C22	115.3 (2)
C2—C12—C11	107.92 (10)	C21—C22—H16	109.5
C2—C12—H5	108.6	C21—C22—H17	109.5
C2—C12—C14	111.51 (10)	C21—C22—H18	109.5
C11—C12—H5	108.6	H17—C22—H16	109.5
C11—C12—C14	111.56 (10)	H18—C22—H16	109.5
C14—C12—H5	108.6	H18—C22—H17	109.5
N2—C13—C11	178.91 (16)	C21—O6—H15	108.3 (19)
			
C1—C2—C3—C4	−2.4 (2)	C12—C11—C10—N1	−173.68 (14)
C1—C2—C3—O3	178.15 (12)	C12—C11—C10—O3	6.6 (2)
C1—O2—C5—C4	−1.4 (2)	C12—C14—C15—C16	178.31 (14)
C1—O2—C5—C9	179.66 (13)	C13—C11—C10—N1	1.0 (2)
C2—C3—C4—C5	0.2 (2)	C13—C11—C10—O3	−178.68 (12)
C2—C3—C4—C6	−179.69 (13)	C14—C12—C2—C1	71.60 (15)
C2—C12—C11—C10	−18.04 (17)	C14—C12—C2—C3	−106.15 (14)
C2—C12—C11—C13	167.21 (11)	C14—C12—C11—C10	104.78 (14)
C3—C2—C1—O1	−176.40 (14)	C14—C12—C11—C13	−69.97 (15)
C3—C2—C1—O2	2.84 (19)	C14—C15—C16—C17	−0.2 (3)
C3—C4—C6—C7	179.99 (14)	C15—C14—C12—C2	47.04 (17)
C3—O3—C10—C11	8.62 (19)	C15—C14—C12—C11	−73.70 (16)
C3—O3—C10—N1	−171.14 (12)	C16—C17—C18—C19	1.2 (3)
C4—C5—C9—C8	−0.7 (2)	C17—C18—C19—C14	−0.1 (2)
C5—C4—C6—C7	0.2 (2)	C18—C17—C16—C15	−1.0 (3)
C5—C9—C8—C7	0.0 (3)	C18—C19—C14—C12	−178.09 (13)
C5—O2—C1—C2	−0.99 (19)	C18—C19—C14—C15	−1.0 (2)
C5—O2—C1—O1	178.31 (12)	C19—C14—C12—C2	−135.97 (13)
C8—C7—C6—C4	−0.8 (3)	C19—C14—C12—C11	103.28 (14)
C9—C5—C4—C3	−179.26 (12)	C19—C14—C15—C16	1.2 (2)
C9—C5—C4—C6	0.6 (2)	C20—O4—C17—C16	−5.0 (3)
C9—C8—C7—C6	0.8 (3)	C20—O4—C17—C18	175.46 (17)
C10—O3—C3—C2	−10.0 (2)	O2—C5—C4—C3	1.79 (19)
C10—O3—C3—C4	170.56 (11)	O2—C5—C4—C6	−178.36 (13)
C11—C12—C2—C1	−165.55 (11)	O2—C5—C9—C8	178.34 (14)
C11—C12—C2—C3	16.70 (17)	O3—C3—C4—C5	179.61 (12)
C12—C2—C1—O1	5.8 (2)	O3—C3—C4—C6	−0.2 (2)
C12—C2—C1—O2	−174.98 (11)	O4—C17—C16—C15	179.48 (15)
C12—C2—C3—C4	175.29 (11)	O4—C17—C18—C19	−179.25 (14)
C12—C2—C3—O3	−4.1 (2)		

### Hydrogen-bond geometry (Å, º)

**Table:**

*D*—H···*A*	*D*—H	H···*A*	*D*···*A*	*D*—H···*A*
N1—H11···N2^i^	0.85 (2)	2.22 (2)	3.062 (2)	172 (2)
N1—H10···O1^ii^	0.81 (2)	2.31 (2)	3.111 (2)	168 (2)
O6—H15···O5^iii^	1.00 (4)	1.67 (4)	2.664 (3)	172 (3)
C15—H6···O5	0.93	2.39	3.251 (2)	154

Symmetry codes: (i) −*x*+2, −*y*, −*z*+1; (ii) *x*+1, *y*, *z*; (iii) −*x*+1, −*y*+1, −*z*+2.
